# Analysis of the Fibrinogen and Neutrophil–Lymphocyte Ratio in Esophageal Squamous Cell Carcinoma: A Promising Blood Marker of Tumor Progression and Prognosis

**DOI:** 10.1097/MD.0000000000001702

**Published:** 2015-10-23

**Authors:** Takaaki Arigami, Hiroshi Okumura, Masataka Matsumoto, Yasuto Uchikado, Yoshikazu Uenosono, Yoshiaki Kita, Tetsuhiro Owaki, Shinichiro Mori, Hiroshi Kurahara, Yuko Kijima, Sumiya Ishigami, Shoji Natsugoe

**Affiliations:** From the Department of Digestive Surgery, Breast and Thyroid Surgery, Field of Oncology, Kagoshima, Japan (TA, HO, MM, YU, YK, SM, HK, YK, SI, SN); Molecular Frontier Surgery, Kagoshima, Japan (TA, YU, SN); and Education Center for Doctors in Remote Islands and Rural Areas, Course of Advanced Therapeutics, Kagoshima University Graduate School of Medical and Dental Sciences, Kagoshima, Japan (TO).

## Abstract

Esophageal squamous cell carcinoma (ESCC) is one of the most aggressive malignancies in gastrointestinal tract cancers and even patients with early ESCC have a high metastatic potential. Difficulties are associated with clinically predicting tumor progression and prognosis based on conventional tumor markers determined from preoperative blood examinations. The aim of the present study was to measure plasma fibrinogen levels and the neutrophil–lymphocyte ratio (NLR) in blood and compare the clinical impacts of their combined values (fibrinogen and neutrophil–lymphocyte ratio score—F-NLR score) and the modified Glasgow Prognostic Score (mGPS) in patients with ESCC.

We classified 238 patients with ESCC based on cut-off values for hyperfibrinogenemia (>400 mg/dL) and high NLR (>3.0) as F-NLR scores of 2 (both of these hematological abnormalities), 1 (one of these abnormalities), or 0 (neither abnormality). We also categorized patients based on cut-off values for high C-reactive protein (CRP) (>0.5 mg/dL) and hypoalbuminemia (<3.8 g/dL) as mGPS of 2 (elevated CRP and hypoalbuminemia), 1 (either elevated CRP or hypoalbuminemia), or 0 (neither elevated CRP nor hypoalbuminemia).

The F-NLR score correlated with the depth of tumor invasion, lymph node metastasis, lymphovascular invasion, tumor size, and stage (all *P* < 0.05). Prognoses among the groups based on the F-NLR score and mGPS significantly differed (all *P* < 0.001). A multivariate analysis identified the depth of tumor invasion, lymph node metastasis, and F-NLR score as independent prognostic factors (*P* = 0.002, *P* = 0.007, and *P* = 0.037, respectively).

The results of the present study showed that the F-NLR score is a promising blood predictor for tumor progression and outcomes in patients with ESCC.

## INTRODUCTION

Esophageal cancer is the tenth most common malignant disease and seventh leading cause of cancer death in Japan, with an estimated 17,497 new patients and 11,746 deaths in 2008.^1^ Esophageal squamous cell carcinoma (ESCC) is the more predominant histological type over esophageal adenocarcinoma in Asian countries, including Japan.^2^ Even patients with superficial ESCC have a high metastatic potential and the 5-year disease-specific survival rate of patients with deep submucosal invasion was previously reported to be 68.6%.^3^ These findings indicate tumor aggressiveness and therapeutic difficulties in patients with ESCC. Furthermore, difficulties are associated with clinically diagnosing the depth of tumor invasion and lymph node metastasis, which are established prognostic factors, based on preoperative examinations. Carcinoembryonic antigen (CEA), squamous cell carcinoma antigen (SCC), and p53 are now clinically used in the management of patients with ESCC. However, these tumor markers are inadequate for identifying subclinical patients with early tumors and predicting disease recurrence.^4^

The Glasgow Prognostic Score (GPS), constructed from C-reactive protein (CRP) and albumin, is an inflammation-based blood marker.^5^ Previous studies reported the clinical impact of GPS as a predictor of tumor progression and prognosis in patients with various malignancies, including ESCC.^6–10^ The modified GPS (mGPS), based on adjusted cut-off values of CRP and albumin, has recently been introduced as a renewed predictive marker of disease outcomes.^11,12^ On the other hand, hyperfibrinogenemia and a high neutrophil–lymphocyte ratio (NLR) have been associated with an advanced tumor stage and poor prognosis in patients with various types of malignancies.^13–18^ Therefore, we proposed a novel prognostic marker based on a combined analysis of plasma fibrinogen values and the NLR in patients with malignant diseases. However, the clinical significance of the combined score determined by plasma fibrinogen levels and the NLR (the F-NLR score) in blood specimens from patients with ESCC has not yet been determined.

The aim of the present study was to assess the relationship between the F-NLR score and clinicopathological factors, including prognosis, in patients with ESCC. We also compared the F-NLR score with mGPS in order to determine its clinical utility as a prognostic marker.

## METHODS

### Patients

Between January 1998 and December 2012, 309 consecutive patients with ESCC underwent esophagectomy with lymphadenectomy at Kagoshima University Hospital (Kagoshima, Japan). The inclusion criteria of this study were as follows: ESCC confirmed by histopathology; patients without endoscopic treatments; patients without palliative resection; patients without preoperative chemotherapy and/or radiotherapy; patients without synchronous or metachronous cancer in other organs; and blood specimens obtained within 2 weeks before surgery. Two hundred thirty-eight patients (210 men and 28 women; age range, 37–87 years; average, 65 years) who fit the criteria were enrolled in the present study. Patients were classified and staged based on the Union for International Cancer Control tumor-node-metastasis (TNM) classification for esophageal carcinoma.^19^Table 1 shows clinicopathological features. All patients were followed-up every 3 to 6 months after surgery by regular clinical diagnostic examinations, such as tumor marker studies (CEA, SCC, and p53), radiography, ultrasonography, and computed tomography at Kagoshima University Hospital. The median follow-up period was 26 months (range, 1–182 months).

**TABLE 1:**
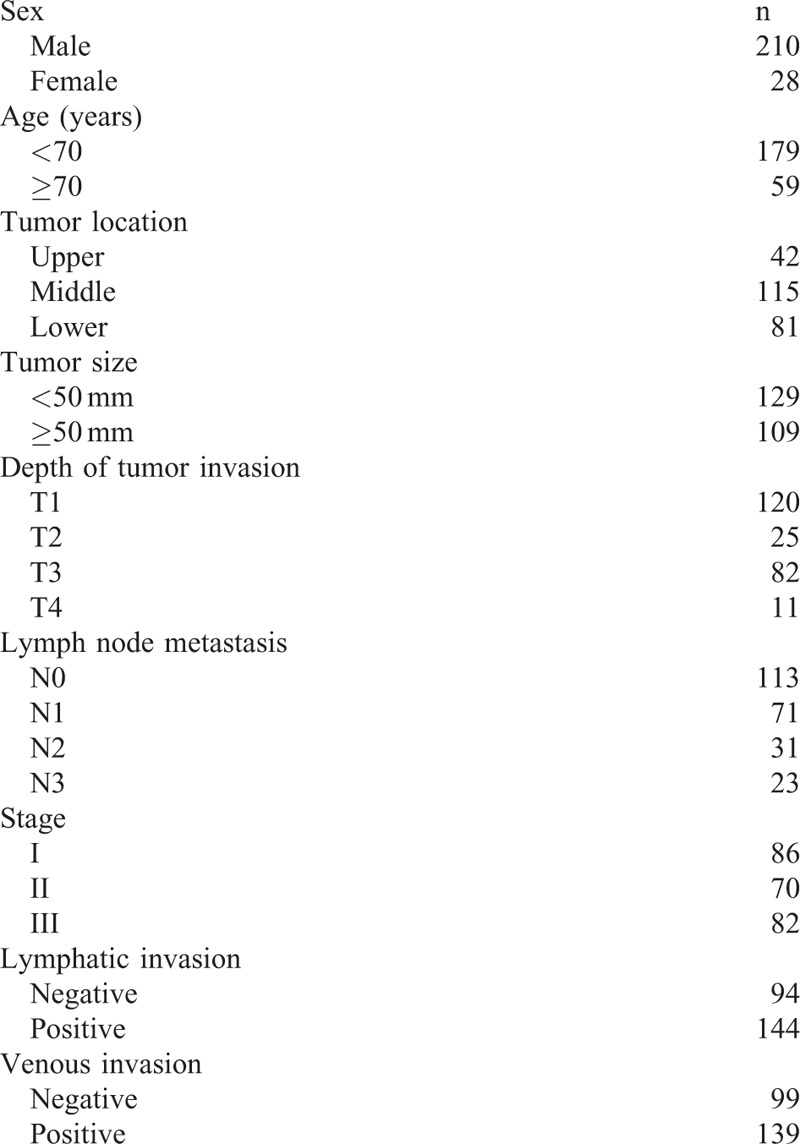
Clinicopathological Features of Patients With Esophageal Squamous Cell Carcinoma (n = 238)

The Ethics Committee at Kagoshima University approved this study and all patients provided written informed consent to the use of their information.

### Blood Analysis for Measurements of Fibrinogen and the NLR

Blood specimens were obtained within 2 weeks before surgery. Plasma fibrinogen concentrations were determined using an STA-R coagulation analyzer (Roche Diagnostics K.K., Tokyo, Japan). Neutrophils and lymphocytes were counted using an XE-2100 automated hematology analyzer (Sysmex Co., Kobe, Japan) and the NLR was then calculated as the neutrophil count divided by the lymphocyte count. CRP and albumin were measured using a JCA-BM automatic analyzer (JEOL Ltd, Tokyo, Japan).

### Statistical Analysis

Differences in the relationships between fibrinogen concentrations or the NLR and categorical clinicopathological features were assessed using the Wilcoxon rank sum test. Relationships between the F-NLR score or mGPS and categorical clinicopathological features were assessed using the Chi-squared test. Differences in survival generated using the Kaplan–Meier method were examined using the log-rank test. Prognostic factors were determined by univariate and multivariate analyses (Cox proportional hazards regression model). All data were statistically analyzed using SAS statistical software (SAS Institute, Inc., Cary, NC). A *P*-value of <0.05 was considered significant.

## RESULTS

### Relationships Between Fibrinogen and Clinicopathological Features

Plasma fibrinogen concentrations ranged between 50 and 640 mg/dL in 238 patients with ESCC. The mean concentration (±SD) of plasma fibrinogen was 369.2 ± 88.9 mg/dL. The mean concentrations (±SD) of plasma fibrinogen in 145 and 93 patients with pT1–T2 and pT3–T4 tumors were 336.4 ± 73.4 and 420.2 ± 87.1 mg/dL, respectively. Hyperfibrinogenemia correlated with deeper tumor invasion (*P* < 0.001). The mean concentrations (±SD) of plasma fibrinogen in 113 and 125 patients with N0 and ≥N1 nodal statuses were 351.6 ± 90.2 and 385.1 ± 85.0 mg/dL, respectively. Patients with lymph node metastasis had significantly higher fibrinogen levels than those without lymph node metastasis (*P* = 0.003). Moreover, plasma fibrinogen concentrations correlated with lymphatic and venous invasion, as well as tumor size (*P* = 0.013, *P* < 0.001, and *P* < 0.001, respectively). The mean concentrations (±SD) of plasma fibrinogen in 86 and 152 patients with stages I and ≥II cancer were 325.0 ± 72.1 and 394.1 ± 87.9 mg/dL, respectively. Categorical stage-related differences in plasma fibrinogen levels significantly differed (*P* < 0.001).

We set the cut-off value of plasma fibrinogen concentrations at 400 mg/dL based on previous findings.^14,15^ All patients were classified into groups with high (>400 mg/dL; n = 76) and low (≤400 mg/dL; n = 162) plasma fibrinogen values. This binary classification of plasma fibrinogen levels was applied in subsequent prognostic analyses.

### Relationships Between the NLR and Clinicopathological Features

The NLR ranged between 0.6 and 10.6 in 238 patients with ESCC. The mean NLR (±SD) was 2.2 ± 1.2. The mean NLR (±SD) in 145 and 93 patients with pT1–T2 and pT3–T4 tumors were 1.9 ± 0.8 and 2.8 ± 1.4, respectively. The NLR correlated with the depth of tumor invasion (*P* < 0.001). The mean NLR (±SD) in 113 and 125 patients with N0 and ≥N1 nodal statuses were 2.0 ± 0.9 and 2.5 ± 1.4, respectively. Patients with lymph node metastasis had significantly higher NLR than those without lymph node metastasis (*P* = 0.001). Furthermore, the NLR correlated with lymphatic and venous invasion as well as tumor size (*P* = 0.003, *P* = 0.002, and *P* < 0.001, respectively). The mean NLR (±SD) in 86 and 152 patients with stages I and ≥II cancer were 1.8 ± 0.8 and 2.5 ± 1.3, respectively. Categorical stage-related NLR significantly differed (*P* < 0.001).

The cut-off value of the NLR was set at 3.0 based on previous findings^16–18^ and all patients were divided into groups with high (>3.0; n = 42) and low (≤3.0; n = 196) NLR. This binary classification of the NLR was applied in subsequent prognostic analyses.

### Prognostic Impact of Fibrinogen or the NLR

**FIGURE 1:**
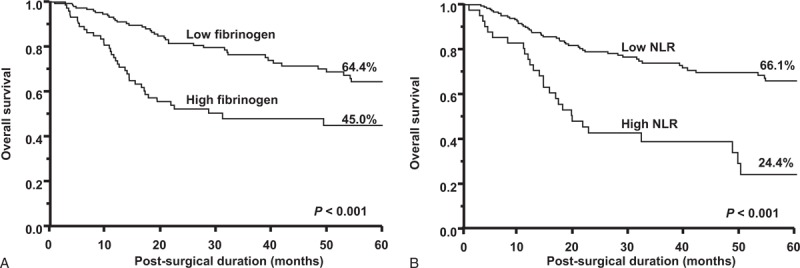
Kaplan–Meier survival curves according to (A) plasma fibrinogen levels and (B) the NLR.

The 5-year survival rate was significantly lower in patients with high fibrinogen values than in those with low fibrinogen values (45.0% vs. 64.4%, *P* < 0.001; Figure 1A). Similarly, the 5-year survival rate was significantly lower in patients with high NLR values than in those with low NLR values (24.4% vs. 66.1%, *P* < 0.001; Figure 1B).

### Relationships Between the F-NLR Score or mGPS and Clinicopathological Features

We classified 238 patients with ESCC as follows: F-NLR score of 2, with hyperfibrinogenemia (>400 mg/dL) and high NLR (>3.0); F-NLR score of 1, with one of these abnormalities; F-NLR score of 0, with neither hyperfibrinogenemia nor high NLR. According to the criteria of the F-NLR score, 147 (61.8%), 64 (26.9%), and 27 (11.3%) patients had F-NLR scores of 0, 1, and 2, respectively. Table 2 shows that the F-NLR score correlated with tumor size, the depth of tumor invasion, lymph node metastasis, stage, and lymphovascular invasion (*P* < 0.001, *P* < 0.001, *P* = 0.003, *P* < 0.001, *P* = 0.002, and *P* < 0.001, respectively).

**TABLE 2:**
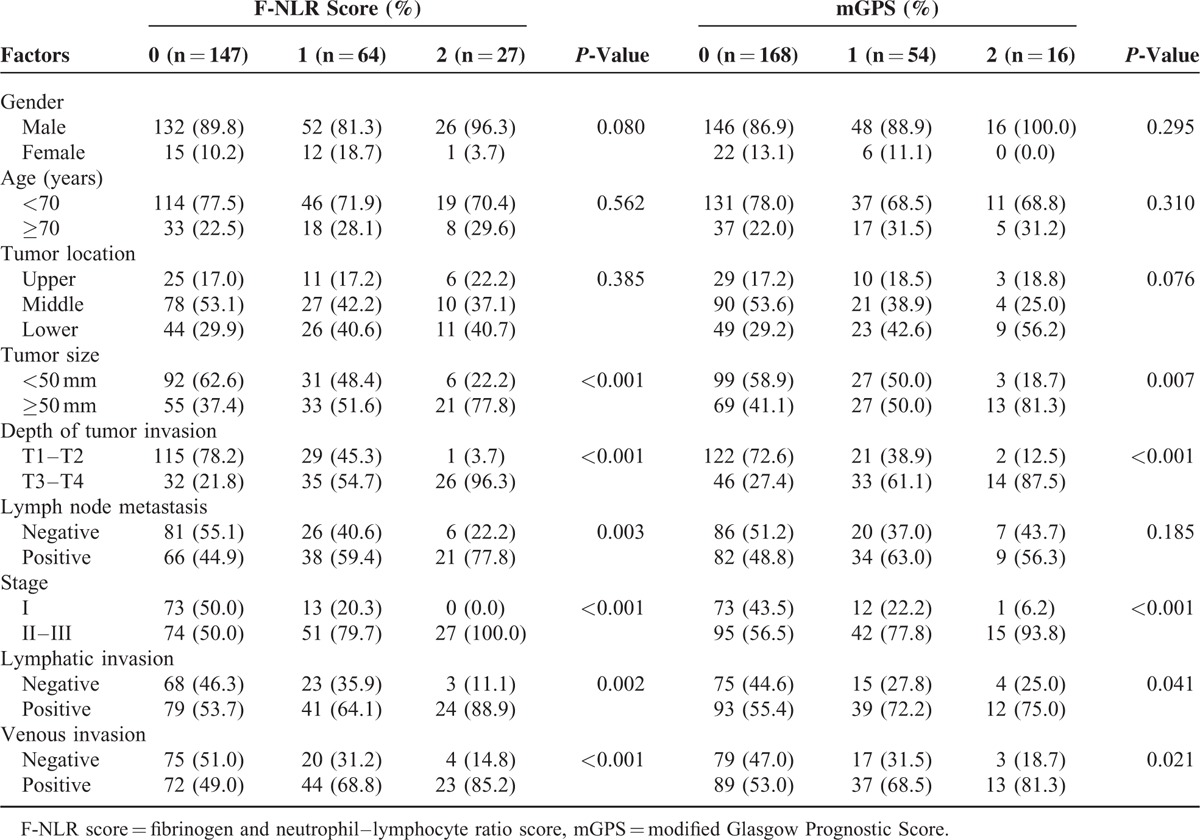
Relationships Between F-NLR Scores or mGPS and Clinicopathological Features (n = 238)

We also categorized patients according to established mGPS criteria as follows: mGPS of 2, with elevated CRP (>0.5 mg/dL) and hypoalbuminemia (<3.8 g/dL); mGPS of 1, with one of these abnormalities; mGPS of 0, with neither elevated CRP nor hypoalbuminemia.^11^ According to the criteria of mGPS, 168 (70.6%), 54 (22.7%), and 16 (6.7%) patients had mGPS of 0, 1, and 2, respectively. Table 2 shows that mGPS correlated with tumor size, the depth of tumor invasion, stage, and lymphovascular invasion (*P* = 0.007, *P* < 0.001, *P* < 0.001, *P* = 0.041, and *P* = 0.021, respectively).

### Prognostic Impact of the F-NLR Score or mGPS

**FIGURE 2:**
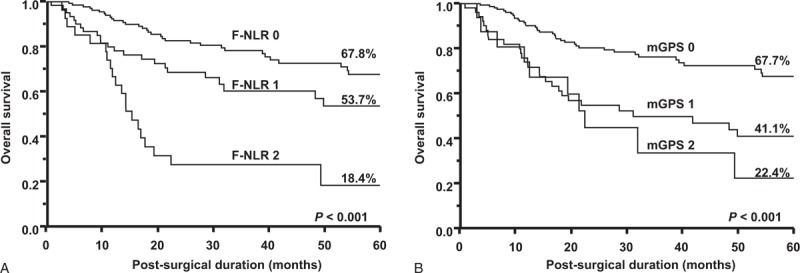
Kaplan–Meier survival curves according to (A) F-NLR scores and (B) mGPS.

Figure 2A shows that the 5-year survival rates of patients with F-NLR scores of 0, 1, and 2 were 67.8%, 53.7%, and 18.4%, respectively. Survival differences based on the status of the F-NLR score were determined to be significant (*P* < 0.001). Figure 2B shows that the 5-year survival rates of patients with mGPS of 0, 1, and 2 were 67.7%, 41.1%, and 22.4%, respectively. Survival differences based on the status of mGPS were significant (*P* < 0.001).

Table 3 shows that the depth of tumor invasion, lymph node metastasis, lymphovascular invasion, F-NLR score, and mGPS correlated with postoperative survival in the univariate analysis (*P* < 0.001, *P* < 0.001, *P* < 0.001, *P* = 0.004, *P* < 0.001, and *P* = 0.013, respectively). The multivariate analysis identified the depth of tumor invasion, lymph node metastasis, and F-NLR score as independent prognostic factors (*P* = 0.002, *P* = 0.007, and *P* = 0.037, respectively) (Table 3).

**TABLE 3:**
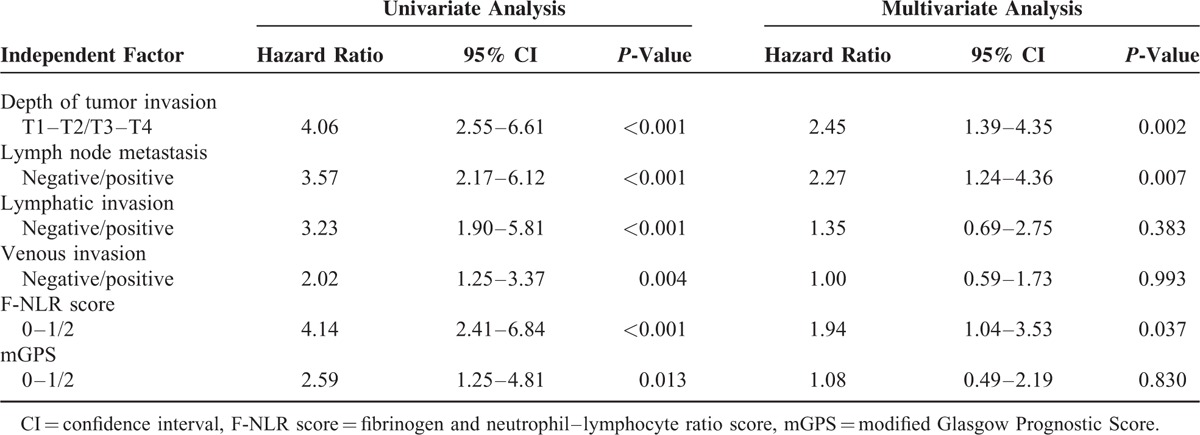
Univariate and Multivariate Analyses of Survival (n = 238)

## DISCUSSION

We herein assessed the clinical significance of fibrinogen levels and the NLR in patients with ESCC. Fibrinogen synthesized by hepatocytes has been shown to play a key role in the coagulation cascade.^20^ Previous studies reported that fibrinogen enhanced tumor proliferation and migration in patients with malignancies.^21,22^ On the other hand, Yamaguchi et al^23^ showed that interleukin-6 produced by cancer cells stimulated the secretion of fibrinogen in patients with lung cancer. Moreover, Sahni et al^24^ demonstrated that fibrinogen was synthesized by cancer cells themselves. A reliable basis for elucidating the relationship between hyperfibrinogenemia and tumor progression has not yet been established. However, these findings indicate that fibrinogen acts as a stimulating factor during tumor progression. Hyperfibrinogenemia exhibits aggressive behavior in various malignant diseases, including ESCC.^13–15^ The present study also found that hyperfibrinogenemia correlated with deeper tumor invasion, increased burden of the lymph node status, the presence of lymphovascular invasion, larger tumor size, and advanced stage in patients with ESCC. Prognostic analyses revealed significantly poorer outcomes among patients with higher fibrinogen levels. These findings suggest fibrinogen is a convenient marker for predicting tumor properties in conventional blood tests.

The systemic inflammatory response has been shown to influence tumor development via multifactorial activities, such as those of vascular endothelial growth factor, interleukin-1, and interleukin-6.^25,26^ Furthermore, the systemic inflammatory response involves an imbalance in the NLR in circulating blood cells.^27^ The NLR has recently been attracting attention as a prominent blood marker that reflects systemic inflammation. Previous studies on the clinical significance of the NLR demonstrated that an elevated NLR was associated with a high risk of recurrence and worse prognosis in patients with several malignancies.^16–18^ The present study also identified more advanced TNM stages and poorer outcomes in patients with high than with low NLR. These results indicated that the NLR is a valuable tool for predicting tumor progression and prognosis based on systemic inflammation in patients with ESCC. The NLR is currently being examined as a predictive marker for responses to chemotherapy or chemoradiotherapy in several types of malignant diseases.^16,28,29^

GPS and mGPS are both established prognostic markers in various malignant neoplasms.^6–12^ The present study also confirmed a close relationship between mGPS and stage or prognosis in patients with ESCC. The major advantage of mGPS is that it can be determined from conventional blood tests without large-scaled instruments. To date, we have searched for novel combined prognostic markers to compete with GPS among various data obtained from blood cell counts as well as biochemical and coagulation tests. We herein demonstrated that the F-NLR score constructed from plasma fibrinogen levels and the NLR correlated with the depth of tumor invasion, lymph node metastasis, lymphovascular invasion, and stage in patients with ESCC. On the other hand, mGPS was not associated with lymph node metastasis (*P* = 0.185). The incidence of lymph node metastasis in patients with an F-NLR score of 2 was higher than that in patients with mGPS of 2 (77.8% vs. 56.3%). Consequently, a relationship may exist between lymph node metastasis and the F-NLR score rather than mGPS. These results suggest that the F-NLR score is superior to mGPS for predicting the presence or absence of lymph node metastasis in patients with ESCC.

We compared the prognostic impact of the F-NLR score and mGPS in the present study. The log-rank test revealed significant survival differences among patients with ESCC classified by F-NLR scores as well as mGPS. The 5-year survival rate of patients with an F-NLR score of 2 was 18.4%. Furthermore, none of the patients with an F-NLR score of 2 had stage I tumors. These results indicated that patients with an F-NLR score of 2 had more malignant tumors than those with F-NLR scores of 0 and 1. Furthermore, the 5-year survival rates of patients with high NLR and mGPS of 2 were 24.4% and 22.4%, respectively. These results demonstrated that patients with an F-NLR score of 2 have a poorer prognosis than those with high NLR or mGPS of 2. If the F-NLR score is preoperatively determined in patients with ESCC, we may be able to discriminate patients with the worst outcomes from all patients. Therefore, a classification system based on the F-NLR score may assist in the selection of patients with ESCC for neoadjuvant systemic chemotherapy or chemoradiotherapy in preoperative management. Moreover, the multivariate analysis selected the F-NLR score, but not mGPS as an independent prognostic factor. The hazard ratio for the F-NLR score was higher than that for mGPS (1.94 vs. 1.08). Therefore, the F-NLR score was found to be superior to mGPS for predicting survival in patients with ESCC.

The present study had several limitations. The median follow-up period was only 26 months. Furthermore, this study was based on a retrospective analysis designed by a single institution. These limitations may have resulted in biases. Accordingly, larger validation studies are needed in order to strengthen the results obtained herein in patients with ESCC.

In conclusion, we suggest that the F-NLR score determined by plasma fibrinogen values and the NLR serves as a prognostic blood marker in patients with ESCC. Our study demonstrated that a close relationship existed between cancer progression and the F-NLR score assessed from preoperative blood specimens. The F-NLR score may be more useful than mGPS as an economical biomarker to plan therapeutic strategies in preoperative patients with ESCC.
